# The effect of T cell aging on the change of human tissue structure

**DOI:** 10.1186/s12979-024-00433-4

**Published:** 2024-04-30

**Authors:** Ling-ling Xu, Xiang Chen, Jing-ping Cheng

**Affiliations:** 1 Medical College, School of Medicine, Wuhan University of Science and Technology, Wuhan, 430065 Hubei China; 2 Department of Gerontology, CR & WISCO General Hospital, Wuhan University of Science and Technology, Wuhan, Hubei, 430080 China

**Keywords:** Aging, Immune aging, T cell aging, Blood vessels, Intestinal flora, Skeletal muscle

## Abstract

The trend of aging of the global population is becoming more and more significant, and the incidence of age-related diseases continues to rise.This phenomenon makes the problem of aging gradually attracted wide attention of the society, and gradually developed into an independent research field.As a vital defense mechanism of the human body, the immune system changes significantly during the aging process.Age-induced changes in the body’s immune system are considered harmful and are commonly referred to as immune aging, which may represent the beginning of systemic aging.Immune cells, especially T cells, are the biggest influencers and participants in age-related deterioration of immune function, making older people more susceptible to different age-related diseases.More and more evidence shows that T cells play an important role in the change of human tissue structure after aging, which fundamentally affects the health and survival of the elderly.In this review, we discuss the general characteristics of age-related T cell immune alterations and the possible effects of aging T cells in various tissue structures in the human body.

## Aging and T cell changes

It is well known that during the aging process, the immune system changes, that is, the disorder and decline of immune system function.In the aging state, the immune system usually shows a relatively continuous state of low activation; When stimulated by the outside world, its dynamic response becomes weaker and the amplitude is reduced, and this combination of chronic inflammatory state and reduced effective defense ability is often referred to as immune aging.

Studies have found that immune aging is associated with increased morbidity and mortality in the elderly [1]。At present, many studies have found relevant factors leading to immune senescence, which are believed to be related to Langerhans cells, dendritic (DC) cells, natural killer (NK) cells, neutrophilic granulocyte function, T cell and B cell function and lineage [2]。Although immune aging affects both the innate and acquired immune systems, the most significant is T-cell immunity [3]。Liu et al. studied various biomarkers associated with human aging in peripheral blood T cells and observed a positive relationship between chronological age and p16 expression as well as telomere shortening, confirming that T cells age with age [4]。With age, the most significant changes are the decrease of the initial T-cell (Tn) library and the increase of the Memory T cell (Tm) library, the decrease of T-cell diversity, the decrease of the number of available TCR_S_, and the decline of T-cell immune response function.In addition, extreme differentiation of memory T cells was observed in the elderly, and co stimulatory molecules such as CD28 and CD27 were no longer expressed, resulting in T cell aging or depletion [5].While aging and depleted T cells exhibit molecular features of aging (such as mitochondrial dysfunction and epigenetic remodeling); Second, the aging of T cells showed signs of DNA damage and short telomeres, and activate signaling pathways associated with aging [6], at the same time a series of factors, these factors are called the senescence associated secretory phenotype (SASP) [7]; With the increase of age, T cells with aging phenotype will continue to accumulate, further promoting immune aging, resulting in a decrease in immune function and an increase in pro-inflammatory function.

At present, it has been found that aging T cells may promote pathological changes in the tissue structure of various systems of the human body through a variety of mechanisms, thereby leading to related diseases and fundamentally affecting the health of the elderly.First, aging T cells continue to produce cytokines that directly promote inflammation; Secondly, aging T cells may not be able to perform the function of monitoring aging, so that they cannot clear the irreversibly damaged cells that become sensory cells, and cannot play the corresponding function in time.In addition, aging T cells can lead to the loss of autoimmune tolerance and secrete cytotoxic substances that directly damage tissues.Finally, aging T cells can also indirectly participate in various changes by regulating intestinal homeostasis.

## T cells and vascular function

In recent years, with the increasing aging trend, the risk of cardiovascular disease is also increasing, which is directly related to the appearance of vascular aging [8].Vascular aging is a degenerative disease of the cardiovascular system with the increase of age, which is usually accompanied by increased stiffness of elastic arteries, decreased compliance, dysfunction of vascular endothelial cells, and weakened ability of vascular repair and regeneration [9].Vascular endothelial cell dysfunction is the early sign of vascular aging, but also the key event.Data from both in vitro and clinical studies suggest that immune system aging affects endothelial cell function and leads to age-related vascular disease [1, 10].In fact, inflammation is an important factor in endothelial cell dysfunction, chronic inflammation can lead to vascular endothelial structure and function changes.Aging of the immune system can promote chronic inflammation, which is a key mechanism of vascular endothelial injury [11].

Trott et al. demonstrated that dysfunctional T cells in older mice are involved in inducing vascular inflammation and associated vascular dysfunction [12], and that these changes may be caused by T cell senescence.Senescent T cells release SASP, including inflammatory cytokines (IL-1β, IL-6), chemokines (GRO1), protease (MMP), vascular endothelial growth factor (VEGF), NO and other signaling molecules, and hormones inducing pro-inflammatory response [13, 14], so that the body can maintain chronic inflammation.The resulting pro-inflammatory microenvironment of blood vessel wall will promote vascular dysfunction, impair cell metabolism, increase cell apoptosis, and lead to the occurrence of corresponding vascular diseases [15]; Secondly, these inflammatory cytokines promote vascular oxidative stress to a certain extent, induce the activation of matrixmetalloproteinase (MMP), degrade vascular wall collagen and elastase, and thus impair the function of vascular endothelial cells, leading to vascular wall remodeling [16]. In addition, the inflammatory factor TNF-a can regulate the damage of endothelial cells through the NF-kB signaling pathway, and TNF-a can stimulate the degradation of IkB protein in cells and activate the NF-kB signaling pathway, resulting in the transcription of genes dependent on NF-kB activation and increased binding to nuclear DNA, thus inducing apoptosis of endothelial cells and accelerating the occurrence of vascular aging [17].Importantly, selective inhibition of NF-κB in the vascular system has been shown to improve blood flow regulation, reduce systemic inflammation, exert beneficial metabolic effects and extend healthy lifespan [18].

On the other hand, studies have found that sirtuins (SIRTs) proteins play an important role in the aging process of blood vessels [19]. Sirtuin (SIRT) or Silence-information regulatory Factor 2 (Sir2) proteins are a class of proteins with nicotinamide adenine dinucleotide (NAD+) -dependent deacetylase activity or ADP-ribosyltransferase activity, which are widely distributed in cells.Currently, it has been found that SIRT1 is the most important factor involved in vascular homeostasis and disease in the SIRT family, and SIRT1 is highly expressed in endothelial cells [19].However, studies have found that SIRT1 protein levels in various aging models gradually decrease with the aging process [20].When T cells age, the expression level of SIRT1 decreases [21].However, downregulation of SIRT1 expression may lead to increased activity of proteins that inhibit cell cycle such as p53, thus inducing endothelial cell senescence [19].

In conclusion, senescent T cells, especially senescent CD8 + T cells, have reduced or depleted expression of the surface co-stimulatory molecule CD28, which is mainly clinically manifested as a significant increase in circulating CD8 + CD28-T cell subsets with aging.Studies have found that from birth to 80 years old, the proportion of CD8 + CD28-T cell subsets in the blood of healthy people increases from 0 to about 60% [22].Compared with CD28 + T cells, CD28-T cells have decreased proliferation capacity and increased expression and secretion of pro-inflammatory cytokines, and play an important role in various age-related cardiovascular diseases [23, 24].Therefore, the detection of CD8 + CD28-T lymphocytes in blood may also serve as an assessment of vascular aging and prediction of cardiovascular risk [25].

## T cells and intestinal mucosal barrier

It is well known that aging is closely related to the integrity of the intestinal barrier [16].The permeability of the intestinal barrier changes with age.Experiments have confirmed that the intestinal permeability of elderly rodents to macromolecules increases, indicating that aging is also related to the deterioration of intestinal barrier function and integrity [26].Evidence shows that T lymphocytes play an important role in maintaining the integrity of the intestinal mucosal barrier [27, 28]。At the same time, immunosuppression and immune imbalance may lead to or aggravate intestinal mucosal barrier dysfunction [29].

Firstly, there are a large number of TRM cells (resident memory T cells), TReg cells (regulatory T cells) and γδT cells in the intestine [23].Gamma-delta T cells, which are most closely related to intestinal mucosal barrier function, produce regenerative factors such as keratinocyte growth factor (KGF) and IGF1 to regulate tissue homeostasis and promote epithelial cell proliferation [30, 31], epithelial integrity is also promoted through the secretion of mediators such as TGFβ1, TGFβ3, and prothymosinβ4 [32].However, it has been found in animal experiments that the proportion of γδT cells is reduced in the gut of elderly mice [33], so we believe that the decline in the number of γδT cells during aging is also an important part of the damage of intestinal mucosal barrier.

Secondly, aging T cells may lead to increased inflammatory response, and a large number of inflammatory factors may lead to dysfunction or excessive apoptosis of intestinal mucosal epithelial cells and impair intestinal mucosal barrier function [34]; In addition, a large number of inflammatory mediators will activate NF-KB (nuclear transcription factor). When NF-KB in intestinal immune cells is activated, downstream signaling molecules will be activated to promote the expression of inflammatory factors, and intestinal immune cells will be activated to cause damage to their mucosal barrier [35].

On the other hand, with the aging of T cells, the immune tolerance of T cells is decreased and the immune regulatory function is impaired, and the ability to recognize beneficial microorganisms in the intestine and immune monitoring and related regulation of harmful microorganisms is decreased, so that the intestinal mucosal barrier is out of balance.Dysfunctional gut microbiota has also been linked to unhealthy aging and age-related chronic inflammatory diseases [36]. Studies have confirmed that T cells promote local IgA responses in germinal centers, thus ensuring tolerance to symbiotic microorganisms [37], but germinal center reactions require a balance of TH17 cells and Treg cells [38, 39]. Their future also will be divided into Tfh cells and T follicular regulatory cells. Recently, iNKT cells have also been shown to control IgA by regulating intestinal flora, as well as regulating the function of intestinal Treg cells [40]. However, dysfunctional germinal center Tfh cell and excessive T follicular regulatory cell activity observed in older mice may affect gut microbiota remodeling during aging [41, 42]. Meanwhile, recent studies have shown that germinal center defects and antigen-specific IgA defects are prone to occur in the elderly [43]. These findings suggest that T cells contribute to the maintenance of a healthy and balanced gut microbiome, and that dysregulation of the gut microbiome can result from T cell aging. In addition, when intestinal flora disorders occur in the body, harmful bacteria increase in the intestine, resulting in dysfunction of intestinal mucosal barrier function and increased permeability, thus activating effector T cells, destroying the balance between them and immunosuppressive regulatory T cells, and further leading to immune function impairment [44]. In short, this loss of host-microbiome symbiosis will aggravate the destruction of the intestinal barrier, promote the spread of intestinal bacterial products throughout the body, cause inflammation, and generate pathological feedback loops that amplify this inflammatory response [45, 46].

In conclusion, T cell aging may affect the immune function, inflammatory state, immune regulation and repair ability of intestinal mucosal barrier, and thus affect the entire intestinal health state.In the elderly population, the continuous decline of intestinal mucosal immune barrier function weakens the intestinal tolerance to its own flora, external infection, malnutrition, dehydration and other conditions, thus providing favorable conditions for the invasion of gastrointestinal pathogens.Therefore, given the role of T cells in regulating intestinal stem cell(ISC) fate and intestinal integrity [47], coupled with their ability to control the gut microbiome, microbiota-T cell interactions have emerged as a potential regulator of health in older adults.Therefore, it is necessary to further study whether the function of aging T cells can be improved by regulating the intestinal microbiota (intestinal microbiota transplantation, oral probiotics, etc.), so as to curb its adverse effects.

## T cells and skeletal muscle

Closely related to age - sarcopenia. It has been reported that immune aging stimulates muscle loss during aging, which can be regarded as a key factor leading to sarcopenia [48]. And T cells play an important role in the middle.

Experiments with T cell-deficient mice and activated spleen T cell mice have shown that the adaptive immune response of T cells to release cytokines to damaged muscles promotes the continuous proliferation of skeletal muscle stem cells, and aging may alter the function of T cell-induced muscle precursor cells (MPC), leading to the occurrence of sarcopenia [49]. Immune aging causes a decrease in the number of muscle stem cells (satellite cells) and transfers muscle stem cells to the fibrogenic phenotype [50], which disrupts muscle regeneration and leads to muscle atrophy. One of the findings suggests that age-related sarcopenia is directly related to muscle stem cell (MuSC) dysfunction, and that high expression of the cell surface marker CD47 (a member of the immunoglobulin superfamily) may lead to decreased proliferation of aging MuSC subpopulations. By blocking CD47 selective polyadenylation mediated by morph or blocking Thrombospondin-1 (THBS1) /CD47 signaling pathway by antibodies, the defects of MuSC regeneration in elderly mice can be overcome, thus improving muscle regeneration in elderly mice [51], which has therapeutic significance. The results of this study provide a new therapeutic target for improving muscle repair ability during aging.

Secondly, aging T cells will secrete inflammatory factors such as senescence associated secretory phenotype (SASP) to accelerate the formation of human inflammatory microenvironment. In the inflammatory microenvironment, excessive cytokines promote the deterioration of skeletal muscle fiber diameter and protein content.It also promotes skeletal muscle metabolic breakdown [52], leading to muscle proteolysis and muscle cell apoptosis [53], and eventually sarcopenia.In addition, T cells also play a role in regulating repair and regeneration after muscle injury. Aging T cells may weaken this regulatory effect, leading to a decline in muscle regeneration.In particular, aging T cells may affect the activity of satellite cells (muscle stem cells), which are essential for muscle regeneration.

On the other hand, animal experiments have found that mice transplanted with probiotics or healthy intestinal microbes have increased skeletal muscle mass, decreased muscle atrophy markers, and enhanced muscle oxidative metabolism, suggesting that intestinal flora plays an important role in skeletal muscle metabolism [54]. Intestinal flora can affect muscle mass by changing amino acid bioavailability, participating in lipid metabolism regulation, and affecting bile acid metabolism [55]. Therefore, T cells can indirectly affect skeletal muscle metabolism by regulating intestinal flora.

In conclusion, aging T cells affect skeletal muscle metabolism through multiple mechanisms such as chronic inflammation, muscle regeneration, muscle remodeling, muscle atrophy, and intestinal flora, leading to impaired muscle function and a decline in muscle mass and strength. So maintaining a healthy immune system and slowing down T-cell aging may help maintain muscle health and function.

## Conclusion and prospect

A healthy immune system seems to be the secret to centenarians’ longevity, which is also contributing to our growing understanding of immune aging.To study the interrelationship between T cell aging and human tissue structure, and to understand the underlying mechanism is an important basis for clinical intervention, hoping to find the key to improve the healthy life span of the elderly.In conclusion, the imbalance of T cell metabolism is the regulator that drives aging-related diseases, so T cell targeted therapy, including clearing senescent T cells and maintaining T cell function, may open up a new therapeutic direction for aging-related diseases.

## Data Availability

No datasets were generated or analysed during the current study.
